# Authors' reply

**DOI:** 10.4103/0971-9261.59614

**Published:** 2009

**Authors:** Mitul Parikh, Ram Samujh, Ravi Prakash Kanojia, K. L. N. Rao

**Affiliations:** Department of Pediatric Surgery, Post Graduate of Medical Education and Research, Chandigarh, India E-mail: rsamujh@yahoo.com

Sir,

I would like to respond to the queries raised.

1. First, the retrospective nature of this work might include some errors and bias in recorded data

Reply: the bias involved in any retrospective study is usually unavoidable

2. Second, the number of subjects in this work might be too few to reach the statistical significance

Reply: the author in the paper had acknowledged that the limitation of the study was a smaller sample size quote ‘The study presented here had a limited sample size and perhaps the results obtained needs to be further validated with more number of patients. Among the identified risk factors for the mortality or the need for laparotomy in those managed with PPD only, we could not identify the threshold level, which can help in decision making’. Unquote.

3. Third, the other important underlying conditions of all neonates including the nutritional status, which is an important indicator in surgery, are not well documented.

Reply: nutritional factor is an independent factor associated with neonates with surgical disease. This was not addressed in this study.

